# Hepatoprotective Effect of *Otostegia persica* Boiss. Shoot Extract on Carbon Tetrachloride-Induced Acute Liver Damage in Rats 

**Published:** 2012

**Authors:** Sedighe Nasiri Bezenjani, Iran Pouraboli, Reza Malekpour Afshar, Gholamabbas Mohammadi

**Affiliations:** a *Department of Biology, School of Sciences, Shahid Bahonar University of Kerman, Kerman, Iran. *; b *Research Center of Physiology, Kerman University of Medical Sciences, Kerman, Iran. *; c *Department of Biochemistry, Medical School of Afzalipour, Kerman University of Medical Sciences, Kerman, Iran*.

**Keywords:** *Otostegia persica*, Hepatoprotective, Carbon tetrachloride, Histopathology, MDA, GSH

## Abstract

In this study, the hepatoprotective effect of the methanol extract of aerial parts (shoot) from *Otostegia persica *Boiss (Golder) was investigated against the carbon tetrachloride (CCl4)-induced acute hepatotoxicity in male rats. Liver damage was induced through the oral administration of 50% CCl4 in liquid paraffin (2.5 mL/Kg bw, per os) 60 min after the administration of the methanol extract of *O. persica *shoot (in 200, 300, 400 mg/Kg bw doses) and assessed using biochemical parameters (plasma and liver tissue malondialdehyde (MDA), transaminase enzyme levels in plasma [aspartate transaminase (AST), alanine aminotransferase (ALT)] and liver glutathione (GSH) levels). Results show that the methanol extract of *O. persica *shoot is active at 300 mg/Kg (per os) and it possess remarkable antioxidant and hepatoprotective activities. Additionally, histopathological studies verified the effectiveness of this dose of extract in acute liver damage prevention.

## Introduction

Liver, which is involved in almost all of the biochemical pathways in the body, plays a vital role in maintaining, performing and regulating its homeostasis. Therefore, a healthy liver is necessary for the health and well-being. Unfortunately, liver is often abused by environmental toxins, poor eating habits and alcohol and medications and over the counter drug use, which can damage and weaken the liver and eventually lead to hepatitis, cirrhosis and other liver diseases (1). Modern medicine has little to offer to alleviate hepatic diseases and there are not many drugs available to treat liver disorders. Hence, many folk remedies of plant origin have been evaluated according to their possible hepatoprotective effects against the liver damage in experimental animals. Silymarin is a polyphenolic component isolated from the fruits and seeds of *Silybum marianum *(2, 3). It restores the GSH content and facilitates the ATPase activity and promotes RNA polymerase I in hepatocytes (4). Flavonolignans isolated from silymarin are known to lead to regeneration of liver tissue (5) and hepatic membrane stabilization response (6). Silymarin has a great potency for hepatoprotection against toxic agents like CCl4 and were used here as a reference drug.

CCl4-induced hepatotoxicity model is frequently used to investigate the hepatoprotective effects of drugs and plant extracts. The changes associated with CCl4-induced liver damage are similar to that of acute viral hepatitis (7).

The family Lamiaceae is one of the largest and most distinctive families of flowering plants with about 220 genera and almost 4000 species worldwide (8). Many biologically active essential oils have been isolated from various members of this family so far. The genus of *Otostegia *is a member of this family which is comprised of 20 species that are distributed over the east of Asia, from them *Otostegia persica *(Burm.) Boiss (*O. persica *) locally called «Golder» is endemic to south of Iran. This is a spiny shrub plant, with about 1.5 m height and with rectangular woody stems. Its leaves are opposite on stems with short petiole and obovate blade and covered with dense white hairs. Flowers have funnel-shaped calyx with longitudinal ridges and bilabiate white corolla with hairy upper lip (9).

The flowers of the plant are widely used as an additive to yoghurt, butter, milk and meat. It has also been used in Iranian traditional medicine as analgesic in toothache and arthritis. Hydroalcoholic extract of *O. persica *alleviates the morphine withdrawal syndrome (10). *O. persica *extracts (methanolic, chloroform and hexane) showed antimicrobial activities against the Gram-positive strains (11). The aqueous extract of the aerial parts of the plant has been used as antispasmodic, antihistaminic and antiarthritic (12). Phytochemical studies on this plant resulted in the isolation and characterization of geraniol, eugenol, ceryl alcohol, hentriacontane, caffeic acid, p- hydroxybenzoic acid, *β*-sitosterol, *β*-sitostery acetate, *β*-amyrin, campesterol and stigmasterol (13). Oral administration of ethanol extract of *O. persica *for 21 days showed antidiabetic effect in rats (14). It has been reported that its ethanolic extract has anti-glycation property which belongs to the known compound 3, 7-dihydroxy-4’, 6, 8-trimethoxy-flavone (15). It has strong antioxidant property and our recent studies indicated that the methanolic extract of its aerial parts has anti-diabetic effect through the stimulation of insulin release and pancreas tissue improvement (16, 17). In addition, it can decrease the hepatic dysfunction originated from diabetes mellitus (18). In this study, we aimed to evaluate the hepatoprotective effect of the methanol extract of *O. persica *shoot on liver injury model in rats.

## Experimental

*Plant material extraction procedure*

The aerial parts of the *O. persica *were collected from Jiroft, Kerman, southeastern of Iran, taxonomically identified and approved by Dr. SM. Mirtadzaddini, Biology Department of Shahid Bahonar University of Kerman (voucher number : 40642, deposited in : Herbarium of Tehran University, director: Dr. F. Attar). The *O. persica *was powdered in an electrical grinder. The extraction was carried out through the maceration of dry plant powder in methanol 80% for 48 h at room temperature. Then, it was submitted to the extraction with methanol by soxhlation. After the extraction, methanol was evaporated by rotary evaporator at 40-50ºC and was dried using a freeze-dryer at - 50ºC. The yield of extraction was 10%. The extract was prepared in distilled water before use.

*Laboratory animals*

Adult male Wistar rats with an average weight of 200-220 g were used in this assay. They were purchased from the animal breeding laboratories of Pasteur Institute (Tehran, Iran) and had free access to food and water, and were maintained in a controlled temperature (24 ± 2ºC) and light cycle (12 h light and 12 h dark).

*Experimental design*

The animals were divided into 6 groups, each consisting of 6 rats orally treated with 50% CCl4 in liquid paraffin (2.5 mL/Kg bw, per os) 60 min after the administration of *O. persica *methanol extract (in 200, 300, 400 mg/Kg bw doses), Legalon which contained 70% silymarin (420 mg/Kg bw) as a reference drug and 0.5 mL distilled water. One group of normal (untreated) rats was also used in our study. Throughout the experiments, local ethical guidelines were considered for taking care of laboratory animals. Twenty-four hours after the CCl4 administration, rats were sacrificed by overdose of diethyl ether and blood samples were withdrawn, collected in heparinized tubes and were centrifuged at 3000 × g for 10 min to obtain plasma. Plasma samples were used to determine the lipid peroxidation level as well as to test the aspartate aminotransferase (AST) and alanine transaminase (ALT) activities. On the other hand, the liver of each rat was promptly removed and used to determine the tissue levels of malondialdehyde (MDA) and glutathione (GSH).

*Biochemical assays*

Pars Azmoon standard kits and RA-1000 Autoanalyzer were used to measure the AST and ALT activities in plasma. The methodology described by Kurtel *et al*. (19) was used to determine the plasma lipid peroxidation level. Besides, rats were sacrificed using diethyl ether to determine the lipid peroxidation in liver tissue. The liver of each rat was immediately excised and chilled in ice-cold 0.9% NaCl and then perfused via the portal vein with ice-cold 0.9% NaCl. After washing with 0.9% NaCl, the method of Ohkawa *et al. *(20) modified by Jamall and Smith (21) was used to determine the lipid peroxidation in tissue samples. The evaluation of cellular GSH in liver tissue was determined by Sedlak and Lindsay method (22).

*Histopathological studies*

For the histopathological study, the livers of six animals in each group were immediately removed and the tissues were fixed in 10% formalin for a period of at least 24 h. The paraffin sections were then prepared (Automatic Tissue Processor, Lietz, 1512) and cut into 5 μm thick sections in a rotary microtome. Thereafter, the sections were stained with haematoxylin-eosin dye and mounted in Canada balsam. The histopathological slides were examined and photographs were taken with a photomicroscope. Histological damage was expressed using the following score system: Ø: absent; ⊥: minimal; +: mild; + +: moderate; + + +: severe.

*Statistical analysis*

The obtained data were analyzed by one-way ANOVA followed by the Tukey’s post-hoc test and p < 0.05 was considered statistically significant.

## Results and Discussion

The effects of the methanol extract of *O. persica *on biochemical parameters of rats intoxicated by carbon tetrachloride (CCl4) were evaluated in this study. CCl4 was found to cause several fold increases in plasma AST (2561.82%) and ALT (3206.53%) levels (Table 1). 

**Table 1:** Effect of methanol extract of *Otostegia persica *on plasma transaminase enzyme levels against the CCl4-induced liver damage

**Materials**	**Dose** **(mg/kg)**	**ALT** **IU/L (mean ± S.E.M)**	**% change** **a**	**AST** **IU/L (mean ± S.E.M)**		**% change** **a**
Control		99.5 ± 3.8	-	165 ± 12.8	-	
CCl4b	2.5c	3290 ± 215.8***	+ 3206.53	4392 ± 129.5***		+ 2561.82
Legalond	420	1434 ± 178.5***	– 56.41	2533 ± 178.5***		– 42.33
Extractd	200	3035 ± 122.3	– 7.75	4119 ± 191.6		– 6.21
	300	2140 ± 249.2**	– 34.95	2996 ± 104.5***	– 31.78	
	400	2857 ± 149.5	– 13.16	3883 ± 49	– 11.59	

a (+) represents percentage of increase and (–) represents decrease in each value compared with either control or CCl4. b Compared with control. c The dose unit is mL/kg. d Compared with CCl4 as hepatotoxin. ** p < 0.01 significant from control or CCl4. *** p < 0.001 significant from control or CCl4

Moreover, the liver (117.21%) and plasma (345.77%) lipid peroxidation levels were increased significantly in CCl4-treated group compared with those in the normal group which has been evidenced by MDA determination. However, the content of GSH in the liver was decreased in CCl4-treated group (51.5%). The plasma (26.6%) and liver (22.96%) MDA level, as well as the plasma ALT (34.95%) and AST (31.78%) were significantly reduced in rats that received a dose of 300 mg/Kg of the methanol extract of the aerial parts of *O. persica*. On the other hand, the content of GSH in the liver tissue was increased significantly (52.5%) by administering 300 mg/Kg of the extract (Tables 1, 2). 

**Table 2:** Effect of methanol extract of *Otostegia persica *on plasma and liver MDA and liver GSH against the CCl4-induced liver damage

**Material**	**Dose** **(mg/kg)**	**Plasma MDA level** **nmol/mL plasma**	**% change** **a**	**Liver MDA level** **nmol/g liver**	**% change** **a**	**Liver GSH level** **μmol/g liver**	**% change**
Control	-	0.673 ± 0.02	-	313 ± 17.4	-	114.32 ± 5.17	-
CCl4b	2.5c	3 ± 0.07***	+ 345. 77	679.86 ± 14.9***	+ 117.21	55.45 ± 3.7***	– 51.5
Legalond	420	1.9 ± 0.35**	– 36.67	445.26 ± 34.4**	– 34.5	91.63 ± 6.56***	+ 65.2
Extractd	200	2.9 ± 0.07	– 3.3	627.4 ± 44.9	– 7.72	62.47 ± 2.38	+ 12.7
	300	2.2 ± 0.24*	– 26.6	523.78 ± 31.5*	– 22.96	84.55 ± 7.2**	+ 52.5
	400	2.8 ± 0.03	– 6.66	602.03 ± 52.1	– 11.45	67.06 ± 3.85	+ 20.9

Results are expressed as mean ± SEM. a (+) represents the percentage of increase and (-) represents the decrease in each value when compared with either control or CCl4. b Compared with control. cThe dose unit is mL/Kg. d Compared with CCl4 as hepatotoxin.*p < 0.05 significant from control or CCl4. **p < 0.01 significant from control or CCl4.*** p < 0.001 significant from control or CCl4

**Figure 1:**
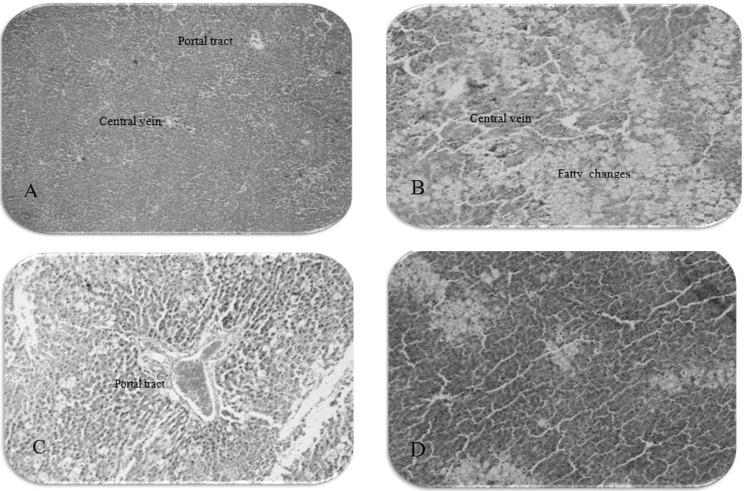
The photomicrographs of liver section from rats: (A) normal control group (10 × 4), (B) received CCl4: liquid paraffin (10 × 10), (C) received Legalon + CCl4 (10 × 10), (D) received extract 300 mg/Kg + CCl4 (10 × 10

Histopathological examination of the liver sections confirmed that the normal liver architecture was damaged with CCl4 administration. However, the pretreatment of methanol extract at 300 mg/Kg significantly lessened the severity of histopathological injury compared with the CCl4 group (Table 3 and Figure 1).

**Table 3:** Histopathological changes in the liver of rats

**Microscopic observation**	**Control**	**CCl4**	**Legalon**	**Extract** **200 mg/kg**	**Extract** **300 mg/kg**	**Extract** **400 mg/kg**
Degeneration in hepatocytes (fatty and hydropic changes)	Ø	+ + +	⊥	+ +	+	+ +
Degeneration in hepatic cords	Ø	+ +	+	+ +	+	+
Deformation in hepatocytes	Ø	+	⊥	+	+	+
Focal necrosis	Ø	+	Ø	⊥	Ø	Ø
Congestion in central vein	⊥	+ +	+	+ +	+	+ +
Congestion in sinusoids	Ø	+	+	+	+	+
Infiltration of lymphocytes	+	+ + +	+	+ + +	+	++
Kupffer cells proliferation	Ø	+	Ø	⊥	⊥	⊥
Bleeding area in hepatic lobes	Ø	Ø	Ø	Ø	Ø	Ø

Ø: absent; ⊥: minimal; +: mild; + +: moderate, + + +: severe

Our results indicate that the methanol extract of *O. persica *exerts hepatoprotective properties against CCl4-induced liver damage. CCl4 is a well-known hepatotoxin and the exposure to this chemical is known to induce the oxidative stress and cause the liver injury through free radicals formation (23). The changes associated with CCl4-induced liver damage are similar to those of acute viral hepatitis (7). In accordance with our findings, it has been shown that the liver of CCl4-intoxicated rats has been exerted the massive fatty change, gross necrosis, broad infiltration of lymphocytes and kupffer cells around the central vein and loss of cellular boundaries (24-28). CCl4-induced hepatotoxicity is believed to include two phases. The initial phase involves the metabolism of CCl4 by cytochrome P450, which leads to the formation of free radicals (CCl3●, CCl3OO●) and lipid peroxidation (29). The second step involves the activation of kupffer cells, probably through free radicals. The activation of kupffer cells is accompanied by the production of proinflammatory mediators (30). As a result of the hepatic injury, the altered permeability of the membrane causes the enzymes from the cells to be released into the circulation which damages the hepatic cells, as shown through the abnormally high level of serum hepatospecific enzymes. Free radicals also affect the antioxidant defense mechanisms, reduce the intracellular concentration of GSH and decrease the activity of SOD and CAT. Lipid peroxidation is a chain reaction that involves the oxidation of polyunsaturated fatty acids in membranes induced by free radicals and is an indicator of oxidative cell damage. Direct measurement of oxidative stress in humans is difficult since the active oxygen species and free radicals are extremely short-lived (31). Instead, products of the oxidative process are measured. The elevation of MDA levels, which is one of the end products of lipid peroxidation in the liver, and the reduction of hepatic GSH levels are important indicators in CCl4-intoxicated rats (32). Glutathione exists in reduced (GSH) and oxidized (GSSG) states. GSH can be regenerated from GSSG through the enzyme glutathione reductase. In healthy cells and tissues, more than 90% of the total glutathione pool is in the reduced form (GSH) and less than 10% exists in the disulfide form (GSSG). An increased GSSG-to-GSH ratio is considered as the indicative of oxidative stress (33).

Two compounds of *O. persica *methanol extract which were separated by column and paper chromatography showed significant antioxidant activities compared with butylated hydroxyl anisole (BHA) and alpha tocopherol. These active compounds were identified as morin and quercetin (34). It is thought that antioxidants play a significant role in protecting the living organisms from the toxic effects of chemical substances such as CCl4 and carcinogens (35). Morin has been shown to act as a potent antioxidant (36), xanthine oxidase inhibitor (37) and modulator of lipoxygenase and cyclooxygenase activities in the arachidonic acid cascade (38). Morin prevents acute liver damage via inhibiting the production of TNF-*α*, IL-6 and iNOS (39). Besides, Quercetin, a natural antioxidant, reveals its antioxidant properties through inhibiting the lipid peroxidation via blocking the enzyme xanthine oxidase (40), and directly scavenging hydroxyl, peroxy and superoxide radicals (41). Quercetin also potentiates an antioxidative defense mechanism through increasing the absorption of vitamin C (42) and inhibiting the structural damage to the proteins (43).

## Conclusion

The methanol extract of *O. persica *has protective effect against the acute liver damage and hepatoprotective mechanisms of this extract on CCl4-induced acute liver damage might be due to the decreased lipid peroxidation (decreased MDA level and increased content of GSH). More studies are needed to determine further mechanisms involved in the hepatoprotective effects of this plant.

## References

[1] Subramonium A, Pushpangadan P (1999). Development of phytomedicines for liver diseases. Indian J. Pharmacol.

[2] Wagner H, Diesel P, Seitz M (1974). Chemistry and analysis of silymarin from Silybum marianum Gaertn. Arzneimittelforschung.

[3] Hahn G, Lehman HD, Kurten M, Uebel H, Vogel G (1968). On the pharmacology and toxicology of silymarin, an antihepatotoxic active principle from Silybum marianum Gaertn (L.). Arzneimittelforschung.

[4] Sonnenbichler J, Mattersberger J, Rosen H (1976). Stimulation of RNA synthesis in rat liver and isolated hepatocytes by silybin, an antihepatotoxic agent from Silybum marianum L. Gaertn. H-S. Z. Physiol. Chem..

[5] Shaker E, Mahmoud H, Mnaa S (2010). Silymarin, the antioxidant component and Silybum marianum extracts prevent liver damage. Food Chem. Toxicol..

[6] Basiglio CL, Sanchez Pozzi EJ, Mottino AD, Roma MG (2009). Differential effects of silymarin and its active component silibinin on plasma membrane stability and hepatocellular lysis. Chem. Biol. Interact.

[7] Rubinstein D (1962). Epinephrine release and liver glycogen levels after carbon tetrachloride administration. Am. J. Physiol.

[8] Hedge IC (1986). Labiatae of South-west Asia: diversity, distribution and endemism. P. Roy. Soc. Edinb.

[9] Rechinger KH (1982). Flora Iranica. Akademiche Druk-u.

[10] Hajhashemi V, Rabbani M, Asghari G, Saravi Z (2004). Effects of Otostegia persica on morphine withdrawal syndrome in mice. Iranian J. Pharm. Res.

[11] Asghari G, Nourallahi H, Havaie SA (2006). Antimicrobial activity of Otostegia persica Boiss extracts. Pharm. Sci.

[12] Ghahraman A (1996). Color Atlas of Iranian Flora.

[13] Ayatollahi SAM, Kobarfard F, Asgarpanah J, Ahmed Z (2007). Chemical constituents from Otostegia persica. J. Chem. Soc. Pak.

[14] Ebrahimpour MR, Khaksar Z, Noorafshan A (2009). Antidiabetic effect of Otostegia persica oral extract on streptozotocin-diabetic rats. Res. J. Biol. Sci.

[15] Ayatollahi SAM, Kobarfard F, Asgarpanah J, Choudhary MI (2010). Antiglycation activity of Otostegia persica (Burm.). Boiss. Afr. J. Biotechnol.

[16] Hedayati M, Pouraboli I, Pouraboli B (2010). Effect of methanolic extract of Otostegia persica on serum levels of glucose and lipids in type I diabetic male rats. IJEM.

[17] Hedayati M, Pouraboli I, Pouraboli B, Dabiri SH, Javadi A (2011). Effect of Otostegia persica extract on serum level of glucose and morphology of pancreas in diabetic rats. Koomesh J. Semnan Univ. Med. Sci.

[18] Hedayati M, Pouraboli I, Mir tajaddini M (2011). The effect of methanolic extract of Otostegia persica on serum levels of glucose and liver function enzymes in streptozotocin -induced diabetic male rats. J. Rafsanjan Univ. Med. Sci.

[19] Kurtel H, Granger DN, Tso P, Grisham MB (1992). Vulnerability of intestinal interstitial fluid oxidant stress. Am. J. Physiol.

[20] Ohkawa H, Ohishi N, Yagi K (1979). Assay for lipid peroxides in animal tissues by thiobarbituric acid reaction. Anal. Biochem.

[21] Jamall IS, Smith JC (1985). Effects of cadmium on glutathione peroxidase, superoxidedismutase, and lipid peroxidation in the rat heart a possible mechanism of cadmium cardiotoxicity. Toxicol. Appl. Pharmacol.

[22] Sedlak J, Lindsay RH (1968). Estimation of total protein-band and nonprotein sulfhydryl group in issue with Ellmann,s reagent. Anal. Biochem.

[23] Manna P, Sinha M, Sil PC (2006). Aqueous extract of Terminalia arjuna prevents carbon tetrachloride induced hepatic and renal disorders. BMC Comp. Altern. Med.

[24] Sreelatha S, Padmab PR, Umadevia M (2009). Protective effects of Coriandrum Sativum on carbon tetrachloride– induced hepatotoxicity in rats. Food Chem. Toxicol.

[25] Jain NK, Lodhi S, Jain A, Nahata A, Singhai AK (2011). Effects of Phyllantus acidus (L.) skeels fruit on carbon tetrachloride- induced acute oxidative damage in livers of rats and mice. J. Clin. Integr. Med.

[26] Song SZ, Choi YH, Jin GY, Li GZ, Yan GH (2011). Protective effect of Cornuside against carbon tetrachloride-induced acute hepatic injury. Biosci. Biotechnol. Biochem.

[27] Huang GJ, Deng JS, Chiu CS, Liao JC, Hsieh WT, Sheu MJ, Wu CH (2012). Hispolon protects against acute liver damage in the rat by inhibiting lipid peroxidation, proinflammatory cytokine, and oxidative stress and downregulating the expressions of iNOS, Cox-2, and MMP-9. Evid-Based Compl. Alt. Med.

[28] Das S, Roy P, Auddy RG, Mukherjee A (2011). Silymarin nanoparticle prevents paracetamol-induced hepatotoxicity. Int. J. Nanomed.

[29] Kotch RR, Glende EA Jr, Rechnagel RO (1974). Hepatotoxicity of bromotrichloromethane-bond dissociation energy and lipoperoxidation. Biochem. Pharmacol.

[30] Edwards MJ, Keller BJ, Kauffman FC, Thurman RG (1993). The involvement of Kupffer cells in Carbon tetrachloride toxicity. Toxicol. Appl. Pharmacol.

[31] Pryor WA, Godber SS (1991). Noninvasive measures of oxidative stress status in humans. Free Rad. Biol. Med.

[32] Souza MF, Rao VSN, Silveira ER (1997). Inhibition of lipid peroxidation by ternatin, a tetrametoxyflavone from Egletes viscos L. Phytomedicine.

[33] Pompella A, Visvikis A, Paolicchi A, De Tata V, Casini AF (2003). The changing faces of glutathione, a cellular protagonist. Biochem. Pharmacol.

[34] Sharififar F, Yassa N, Shafiei A (2003). Antioxidant activity of Otostegia persica (Labiatae) and its constituents. Iranian J. Pharm. Res.

[35] Sheweita SA, El-Gabar MA, Bastawy M (2001). Carbon tetrachloride changes activity of cytochrome P450 system in the liver of male rats: role of antioxidants. Toxicology.

[36] Wu TW, Zeng LH, Wu J, Fung KP (1994). Morin: a wood pigment that protects three types of human cells in the cardiovascular system against oxyradical damage. Biochem. Pharmacol.

[37] Yu Z, Fong WP, Cheng CH (2006). The dual actions of morin (3,5,7,2›,4›- pentahydroxyflavone) as a hypouricemic agent: uricosuric effect and xanthine oxidase inhibitory activity. J. Pharmacol. Exp. Ther.

[38] Laughton MJ, Evans PJ, Moroney MA, Hoult JR, Halliwell B (1991). Inhibition of mammalian 5-lipoxygenase and cyclooxygenase by flavonoids and phenolic dietary additives. Relationship to antioxidant activity and to iron ion-reducing ability. Biochem. Pharmacol.

[39] Lee HS, Jung KH, Hong SW, Park IS, Lee C, Han HK, Lee DH, Hong SS (2008). Morin protects acute liver damage by carbon tetrachloride (CCl4) in rat. Arch. Pharm Res.

[40] Cheng LE, Breen K (2000). On the ability of four flavonoids, baicilein, luteolin, naringenin, and quercetin to suppress the fentone reaction of the iron-ATP complex. BioMetals.

[41] De Walley CV, Rankin SM, Hoult JR, Jessup W, Leake DS (1990). Flavonoids inhibit the oxidative modification of low density lipoproteins by macrophages. Biochem. Pharmacol.

[42] Vinson JA, Bose P (1998). Comparative bioavailability to humans of ascorbic acids alone or in Citrus extract. Am. J. Clin. Nutr.

[43] Salvi A, Carrapt P, Tillement J, Testa B (2001). Structural damage to protein by macrophages and influence of protein binding. Biochem. Pharmacol.

